# A simple medical device development according to normative values of calf circumference across ages: results from the Italian Longevity Check‐up 7+ (Lookup 7+) project

**DOI:** 10.1002/jcsm.13286

**Published:** 2023-12-06

**Authors:** Anna Maria Martone, Francesca Ciciarello, Vincenzo Galluzzo, Riccardo Calvani, Maria Beatrice Zazzara, Matteo Tosato, Hélio José Coelho‐Junior, Emanuele Marzetti, Francesco Landi

**Affiliations:** ^1^ Fondazione Policlinico Universitario ‘Agostino Gemelli’ IRCCS Rome Italy; ^2^ Department of Geriatrics, Orthopedics and Rheumatology Università Cattolica del Sacro Cuore Rome Italy

**Keywords:** Calf circumference tape, Early diagnosis, Muscle mass, Screening

## Abstract

**Background:**

Wide consensus exists on the notion that low muscle mass is a predictor of negative health‐related events, such as disability, morbidity, and mortality. Indeed, the European Working Group on Sarcopenia in Older People 2 had identified muscle mass as the key component to confirm the diagnosis of sarcopenia. However, the lack of normative values for muscle mass across ages hampers the practical assessment of this important parameter. The aim of the present study was to produce cross‐sectional centile and normative values for calf circumference (a surrogate estimation of muscle mass) across a wide spectrum of ages using a large and unselected sample of community‐dwellers enrolled in the Longevity Check‐up 7+ (Lookup 7+) project.

**Methods:**

This is a cross‐sectional study using the data of Lookup 7+ project, an ongoing study started in June 2015 and conducted in unconventional settings (i.e., exhibitions, malls, and health promotion campaigns). Candidate participants were considered eligible for enrolment if they were at least 45 years of age and provided written informed consent. Calf circumference was measured using an inextensible but flexible plastic tape in a sitting position with the knee and ankle at a right angle and the feet resting on the floor. Normative values for calf circumference from ages 45 to 80 + years were generated.

**Results:**

A total of 11 814 participants were enrolled from 1 June 2015 to 30 September 2022. The mean age of participants included in the analyses was 61.8 years (standard deviation; 10.2 years; range: 45–98 years), and 6686 (57%) were women. Normative values for calf circumference were obtained for men and women, stratified by age groups. Accordingly, a calf circumference tape, with colour bands that demarcate the centiles range into which the patient falls, was created and validated.

**Conclusions:**

Our study established age‐ and gender‐specific centile reference values for calf circumference. The calf circumference tape can be used to easily interpret the assessment in every‐day practice for the early detection of individuals with or at risk of sarcopenia and malnutrition.

## Introduction

Sarcopenia has received increasing awareness by researchers and clinicians, such that it is now considered as a ‘true’ illness with its individual ICD‐10 code.^1^ In 2019, the European Working Group on Sarcopenia in Older People 2 released an updated consensus on the definition and diagnosis of sarcopenia, identifying muscle mass as the domain for the ‘definitive’ diagnosis of sarcopenia.^2^ The age‐related loss of skeletal muscle mass is well recognized as one of the most important risk factors for several negative health‐related outcomes.^3^, ^4^ Not surprisingly, the Global Leadership Initiative on Malnutrition (GLIM) consensus included low muscle mass as one of the criteria for the malnutrition definition.^5^

There are many sophisticated muscle mass and muscle quality evaluation tools—such as bioelectrical impedance analysis, dual X‐ray absorptiometry, computed tomography, magnetic resonance imaging, ultrasound—but primary healthcare system is typically characterized by limited resources. In this context, it is particularly important to look for easier and inexpensive methods to estimate skeletal muscle mass.^6^

When body composition techniques are not accessible, surrogate approaches can be used, including anthropometry assessment (e.g., calf circumference). Such approaches can help health care professionals to identify high‐risk patients, potential targets for early intervention to prevent and/or treat muscle loss. Calf circumference is an anthropometric measure highly correlated with direct and indirect measures of skeletal muscle mass.^7^, ^8^, ^9^ In particular, the World Health Organization (WHO) has proposed the use of calf circumference as a specific marker of muscle mass in older and frail subjects, and research conducted in many European Countries revealed that the use of such measure as a proxy of muscle mass index was more frequent than that of diagnostic imaging, such as dual X‐ray absorptiometry or computed tomography, especially in primary care setting.^9^, ^10^ Likewise, as recommended by the Asian Working Group for Sarcopenia Consensus^11^ and the GLIM Body Composition Working Group,^5^ calf circumference can also be utilized as a screening tool for case‐finding in general populations and different health care settings when body composition techniques are not available.

While calf circumference has many significant qualities as a practical tool for providing an estimate of muscle mass, an important gap remains considering the different cut‐off points suggested, mostly derived from studies in older persons.^12^, ^13^, ^14^ In this perspective, the aim of the present study is to produce cross‐sectional centile values for calf circumference across ages (from 45 to 80+ years), analysing a large and unselected sample of healthy participants enrolled in the Longevity Check‐up 7+ (Lookup 7+) project.

As a second step of our study, we considered that what is really needed is a simple device capable of estimating calf circumference in any adult and old subjects, at any age, without regard for concerns related to inaccuracies arising from unmatched reference populations. Finally, we created and validated a prototype device to simply assess calf circumference centile score without ancillary reference charts or calculators.

## Methods

Data used in the present study are taken from the Lookup 7+ project, an initiative promoted by the Department of Geriatrics of the Fondazione Policlinico ‘Agostino Gemelli’ IRCCS at the Università Cattolica del Sacro Cuore (Rome, Italy).^15^ The Lookup 7+ project was designed and implemented with the aim of encouraging the adoption of healthy lifestyles in the general population. People entering public spaces (i.e., exhibitions and shopping centres) or those adhering to prevention campaigns launched by our institution were enrolled.

The Lookup 7+ protocol was approved by the Ethics Committee of the Università Cattolica del Sacro Cuore (protocol #: A.1220/CE/2011) and is described in detail elsewhere.^15^, ^16^ The manuscript was prepared in compliance with the STrengthening the Reporting of OBservational studies in Epidemiology (STROBE) reporting guidelines for observational studies.

### Study sample

Between 1 June 2015 and 30 September 2022, 14 934 persons were enrolled in different Italian cities participating the national Lookup 7+ project. Participants were considered to be eligible to participate to the Lookup 7+ screening if they were at least 18 years old and gave written informed consent. Exclusion criteria were self‐reported pregnancy, inability to perform the physical performance test (i.e., wheel‐chaired), or inability to give written informed consent.

For the present study, we selected subjects older than 45 years of age (*n* = 11 954). Even though in the Longevity check‐up 7+ database we have data starting from the age of 18‐year‐olds, we decided to do the analysis identifying normative values for calf circumference starting from 45 years of age. This decision was made to obtain a useful screening tool starting from the age that previous scientific evidence has shown to be the beginning of muscle mass decline. In the cleaning database activity, all the potential outliers for the variables of interest were checked. After checking the value with the row data, the correct value was considered. In case of a clear mistake in the row data (i.e., calf circumference of 70 cm), the value was considered as missing data. As a result, the final analysis was conducted on 5119 males and 6695 females (after excluding 140 participants for missing calf circumference data). The general characteristics of the excluded participants did not differ from those with complete data for the variable of interest.

### Data collection

All persons who accepted to participate in the study underwent individual assessment consisting of a brief questionnaire and the measurement of several parameters. In particular, the Lookup 7+ visit was structured to collect the following information and data: informed consent, lifestyle interview (smoking and dietary habits and physical activity), and measurement of body mass, height, and muscle mass and strength.^17^, ^18^ Smoking habit was categorized as current or never/former smoker. Participants who claimed to have quit smoking were still classified as no smokers. Body weight was measured through an analogue medical scale. Body height was measured using a standard stadiometer. Body mass index (BMI) was defined as weight (kilograms) divided by the square of height (metres). Healthy diet was considered as the consumption of at least three portions of fruit and/or vegetables per day.^19^ Regular participation in physical activity was considered as involvement in exercise training at least twice a week.^20^

### Calf circumference

Calf circumference was measured using an inextensible but flexible plastic tape in a sitting position with the knee and ankle at a right angle and the feet resting on the floor.^21^ The calf circumference was measured at the point of maximum circumference on a plane perpendicular to the long axis of the right calf. Subcutaneous tissues were not compressed. The measurement was taken directly on the skin by suitably trained health care professionals. When the calf was not ‘easily available’, the participant was asked to remove the clothes. The values obtained were rounded to the nearest 0.1 cm.

### Statistical analysis

Continuous variables are shown as mean ± standard deviation (SD), categorical variables as frequencies by absolute value and percentage of the total. Descriptive statistics were run to describe demographic and main clinical characteristics of the study population according to age groups and gender. Differences in proportions and means of covariates among age and gender groups were assessed using the Fisher's exact test and *t*‐test, respectively.

We produced gender‐specific cross‐sectional centiles (5th, 25th, 50th, 75th, and 95th) for calf circumference. Accordingly, a simply device—calf circumference tape—was created in which circumferential tape measure (which enables determination of calf circumference in centimetres/millimetres) was overlaid with colour bands that demarcate the centiles score range into which the patient of a given age and gender falls. Finally, to quantify the agreement of calf circumference assessment device between two different assessors, the weighted kappa was calculated with intraclass correlation coefficient. Furthermore, the level of agreement between the calf circumference device and the standard flexible tape was assessed with Pearson's correlation coefficient.

All analyses were performed using SPSS software version 11.0 (SPSS Inc., Chicago, IL, USA).

## Results

The mean age of participants included in the analyses was 61.8 years (SD; 10.2 years; range: 45–98 years), and 6686 (57%) were women. The general characteristics of the study sample according to age and gender are summarized in Table 1. The proportion of smokers, considering only active smokers, decreased progressively with age in both genders (22% among 45‐year‐old subjects vs. 9% among older subjects), with a greater prevalence in men in all age groups. BMI increased with age up to 65 years and slightly declined thereafter. In all age groups, BMI was higher in men than in women (26.5 and 25.1 kg/m^2^, respectively). Healthy diet—defined as the consumption of at least three portions (~400 g) of fruit and/or vegetables per day—was more frequent in older age groups (60% among 45‐year‐old subjects vs. 73% among older subjects), with no significant differences between genders. Finally, the rate of participation in any kind of physical activity for at least two times per week was higher in younger age groups than among older persons (54% among 45‐year‐old subjects vs. 49% among older subjects). For all age groups, the engagement in physical activity was more frequent in men than in women (59% and 52%, respectively).

**Table 1:** General characteristics of the study sample according to age and gender

Characteristics	Age groups (years)								
	45–49	50–54	55–59	60–64	65–69	70–74	75–79	80+	All
**Women**									
Height (cm)	163.3 ± 6.6	163.5 ± 6.6	161.6 ± 6.7	160.5 ± 6.8	158.9 ± 6.9	157.8 ± 6.5	157.1 ± 6.6	156.1 ± 7.2	160.4 ± 7.1
Weight (kg)	64.7 ± 12.5	64.8 ± 12.1	64.2 ± 12.0	64.7 ± 11.0	65.1 ± 10.8	65.4 ± 11.6	64.1 ± 11.2	62.5 ± 10.6	64.6 ± 11.6
BMI (kg/m^2^)	24.2 ± 4.5	24.5 ± 4.3	24.5 ± 4.4	25.1 ± 4.2	25.8 ± 4.1	26.2 ± 4.5	26.0 ± 4.6	25.6 ± 4.2	25.1 ± 4.4
Healthy diet	550 (65)	820 (67)	834 (73)	779 (76)	772 (80)	596 (78)	405 (78)	212 (71)	4968 (73)
Physically active	433 (51)	660 (54)	598 (52)	553 (54)	530 (55)	403 (53)	252 (42)	136 (46)	3379 (52)
**Men**									
Height (cm)	176.5 ± 6.6	175.9 ± 6.5	174.8 ± 7.0	175.6 ± 7.0	172.6 ± 6.6	170.6 ± 6.6	169.9 ± 6.5	168.9 ± 6.5	175.4 ± 7.1
Weight (kg)	81.0 ± 12.6	81.1 ± 12.4	80.5 ± 12.2	81.1 ± 12.7	80.9 ± 12.5	78.2 ± 11.3	76.5 ± 10.5	75.1 ± 10.1	79.8 ± 12.2
BMI (kg/m^2^)	26.0 ± 3.7	26.1 ± 3.7	26.3 ± 3.6	26.9 ± 3.3	27.1 ± 3.9	26.8 ± 3.5	26.4 ± 3.2	26.3 ± 3.2	26.5 ± 3.4
Healthy diet	330 (55)	425 (54)	500 (56)	458 (60)	503 (70)	515 (75)	351 (79)	235 (75)	3317 (64)
Physically active	352 (58)	473 (60)	532 (60)	450 (59)	398 (56)	417 (61)	259 (59)	167 (54)	3048 (59)

Data are given as number (per cent) for healthy diet and physically active; for all the other variables, means ± standard deviations are reported. Regular physical activity: physical activity at least twice weekly during the past year. Healthy diet: consumption of at least three portions of fruit and/or vegetables per day.

BMI, body mass index.

**Figure 1:**
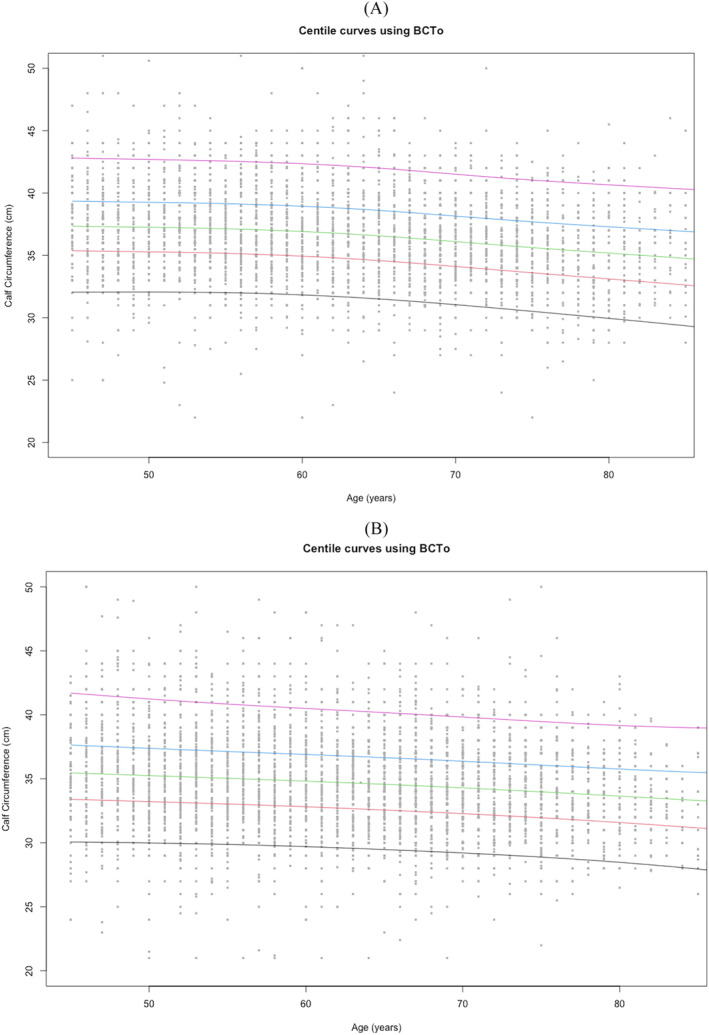
Calf circumference reference percentiles for men (panel A) and women (panel B) aged 45 to 80+ years. The 5th, 25th, 50th, 75th, and 95th percentiles are shown in black, red, green, blue, and purple, respectively.

Normative values for calf circumference (centimetre) in men and women, stratified by age groups, are shown in Tables 1 and 2, respectively. Mean values ± SD and the 5th, 25th, 50th, 75th, and 95^th^ percentiles are reported. Average calf circumference values remain substantially stable from 45 to 60 years old and decline thereafter in both women and men. For all age groups, the calf circumference of men was always higher than that of women. Charts with centile values for calf circumference measurements in the two genders are shown in Figure 1.

**Table 2:** Normative values of calf circumference (cm) in men, stratified by age

Age (years)	Observations	Centiles					Mean (SD)
		5th	25th	50th	75th	95th	
45–49	595	33.0	35.5	37.0	39.0	42.6	37.1 (3.4)
50–54	771	32.8	35.5	37.0	39.0	42.5	37.4 (3.4)
55–59	881	32.0	35.0	37.0	39.0	42.5	37.1 (3.4)
60–64	753	31.5	35.0	36.7	39.0	42.0	36.8 (3.6)
65–69	704	31.2	34.4	36.3	38.6	42.0	36.5 (3.2)
70–74	675	31.0	34.0	36.0	38.0	41.0	35.9 (3.0)
75–79	436	30.0	33.0	35.4	38.0	41.0	35.3 (3.2)
80+	304	30.0	32.5	35.0	37.0	41.0	35.0 (3.4)

**Figure 2:**
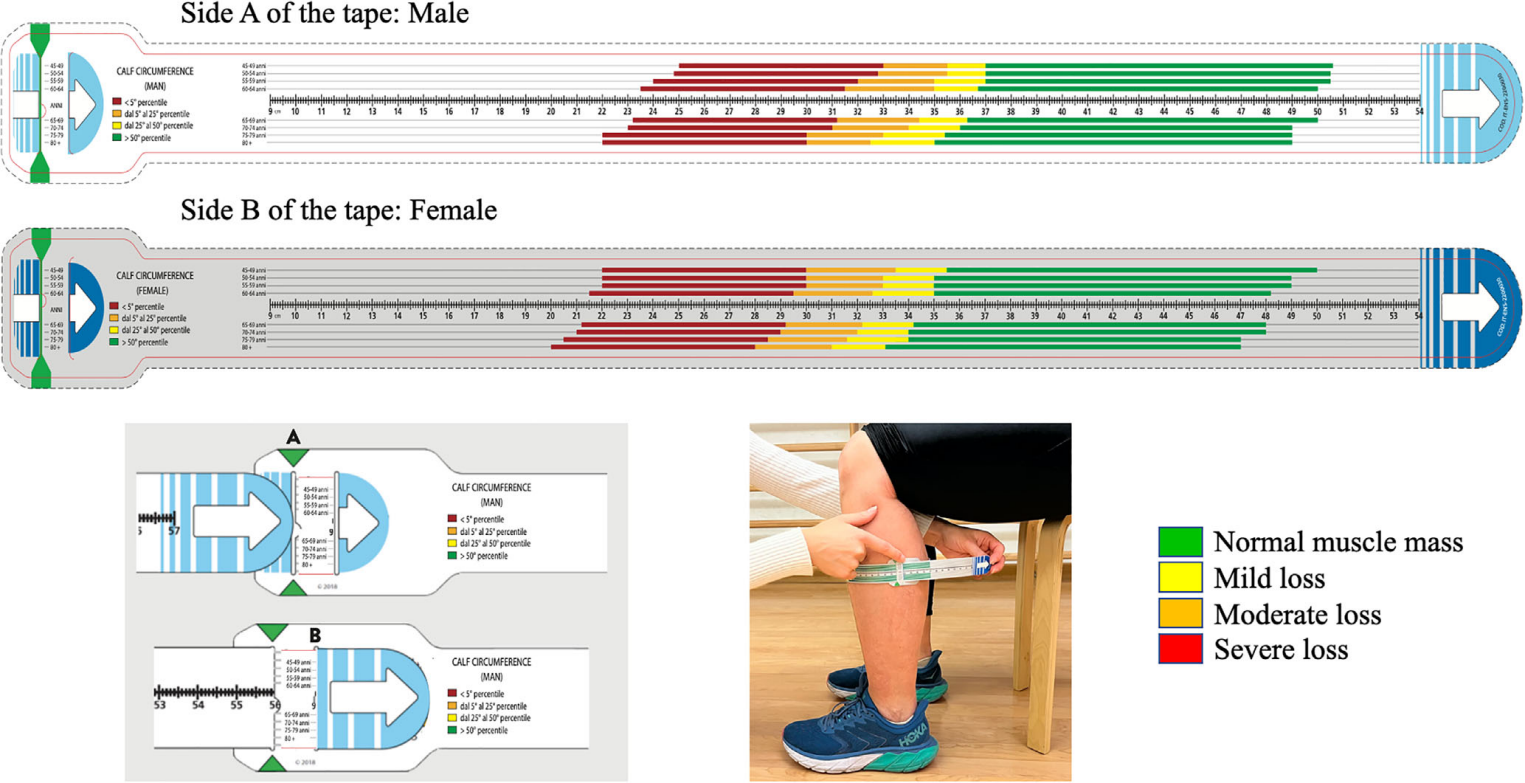
Prototype of calf circumference tape (not drawn to scale) with an enlarged view to explain how the loop is shaped. The green area indicates normal calcircumference, yellow area indicates mild loss, orange area indicates moderate loss, and red area indicates severe loss. The figure shows how to use the tape: (A) select the side of the tape by gender; (B) identifies the point of greatest calf circumference; (C) slide the end of the tape into slit A and back out through slit B to create a loop; (D) the tail end of the tape is pulled until it is snug but does not compress the skin; (E) identify the colour band corresponding to the subject's age. Example of man with normal BMI (23.5 kg/m^2^). Calf circumference: 41.7 cm; age of subject: 58 years; centile‐score in green area (centile greater than 50th) means normal muscle mass. Example of woman with overweight BMI (27.2 kg/m^2^). Calf circumference: 36.5 cm; age of subject: 68 years; subtracting 3 cm, adjusted calf circumference: 33.5 cm; centile‐score in yellow area (centile between 25th and 50th) means mild muscle mass loss.

To help translate these data into clinical practice, *ad hoc* device (Figure 2) depicting the 5th, 25th, and 50th, from age 45 to age 85+, in men and women, was created. The red coloured area indicates values below the 5th centile, the orange values between the 5th and 25th centile, the yellow values between 25th and 50th centile, and green values above the 50th centile.

To test the reliability of the calf circumference tape, the agreement between two different assessors was evaluated. Two assessors separately evaluated 67 subjects (30 males and 37 females, ranging from 45 to 86 years old). The overall reliability between the assessors was almost perfect (intraclass correlation coefficient = 0.88). Finally, agreement between the calf circumference device and the standard flexible tape was assessed with Pearson's correlation coefficient. The value of *R* ^2^ was 0.91, indicating an excellent agreement (Table 3).

**Table 3:** Normative values of calf circumference (cm) in women, stratified by age

Age (years)	Observations	Centiles					Mean (SD)
		5th	25th	50th	75th	95th	
45–49	833	30.0	33.5	35.5	38.0	42.0	35.6 (3.5)
50–54	1201	30.0	33.0	35.0	37.0	41.0	35.1 (3.5)
55–59	1126	30.0	33.0	35.0	37.0	41.0	35.0 (3.6)
60–64	1010	29.5	32.6	35.0	37.0	40.2	34.8 (3.5)
65–69	956	29.2	32.2	34.2	36.5	40.0	34.5 (3.3)
70–74	759	29.0	32.0	34.0	36.5	40.0	34.3 (3.3)
75–79	514	28.5	31.6	34.0	36.0	39.0	33.7 (3.3)
80+	296	28.0	31.0	33.1	35.8	39.0	33.4 (3.3)

## Discussion

Findings from the present study indicate that muscle mass, as assessed by calf circumference, is quite stable in adult life and decline with advancing age. The normative values determined for calf circumference across age groups are functional to the practical interpretation of muscle mass measurement and the definition of thresholds for the screening and case finding of sarcopenia and malnutrition in clinical practice. Because malnutrition has been proposed as the biological substrate of sarcopenia and sarcopenia as biological substrate of physical frailty,^22^ our data may be extremely useful in the early identification of people at risk of incurring negative health‐related events. This implies considerable clinical and public health implications, because preventive interventions against sarcopenia and malnutrition may start from middle age, when decline in muscle mass and strength becomes evident.

According to the European Working Group on Sarcopenia in Older People consensus,^2^ the ‘definitive’ diagnosis of sarcopenia requires an appropriate muscle quantity and quality assessment, which is typically based on high‐cost, time‐consuming, or health facility‐restricted methods. Similarly, according to the GLIM consensus, low muscle mass has been comprised as one of the conditions for the malnutrition definition.^5^ In this context, it is particularly important to look for easier and inexpensive methods to estimate skeletal muscle mass.^6^ In this scenario, anthropometric measurements, mainly calf circumference and mid‐upper arm circumference, have been previous suggested as indirect indicators of skeletal muscle mass.^23^, ^24^ In particular, calf circumference is a validated, low‐cost, no time consuming, easy to reproduce, and commonly available anthropometric measure, whose correlation with appendicular skeletal muscle mass has been previously described to range from good to moderate.^7^, ^12^, ^21^

The present is the first, except for Gonzalez et al.,^25^ large‐scale study to produce normative data for calf circumference across a wide age spectrum in an unselected study sample of Caucasian persons. Based on this unique data, a device depicting the 5th, 25th, 50th, >50th centile, the ‘calf circumference tape’ could be a useful tool to measure muscle mass from age 45, similarly to the paediatric tape widely used to assess malnutrition in children.^26^, ^27^ Hence, the device produced and validated by the present study may become a unique, simple, and rapid tool for the assessment of all persons with or at risk of malnutrition and/or probable sarcopenia in any care setting.

In this regard, it is worth noting that currently available cut‐offs for calf circumference previously suggested are not specific for gender and are not stratified for age and may therefore not be used for the early identification of people who might be at risk for sarcopenia. For instance, a calf circumference value of 34.5 cm in a 45‐year‐old man would be considered normal according to the currently available cut‐offs,^2^ whereas such a calf circumference value would fall between the 5th and 25th centile for age and gender based on our the Lookup 7+ data.

Albeit dealing with a highly relevant issue, our study presents some limitations that need to be discussed. First, normative values for calf circumference were obtained cross‐sectionally. Centiles should therefore not be used to monitor an individual's trajectory of changes in muscle mass over time. Second, the unconventional setting in which the study was conducted might have influenced the results of calf circumference testing. Indeed, although calf circumference was measured according to standard protocols, people who decided to participate in the study were involved—before being assessed—in a variety of activities, such as walking, carrying bags, and eating. Such activities could have influenced the assessment. Limitations also include the lack of information about specific medical conditions (e.g., osteoarthritis, osteoporosis, and other musculoskeletal and neurological disorders impacting muscle strength generation). However, it may reasonably be excluded that acute and/or severe illnesses were present at the time of evaluation. Furthermore, the study population was conducted in Italy including only Caucasian persons; thus, our findings and charts may not be applicable to other ethnic groups. Finally, in this study the influence of body mass index (BMI) on age and gender calf circumference variation has not been considered in the creation of the device. However, transferring BMI‐adjusted calf circumference values into a single device or creating multiple devices based on different BMI values is not really feasible. Obviously, when evaluating the calf circumference of overweight or obese (BMI > 25.0 kg/m^2^) subjects, correction factors need to be considered. In this respect, it important to highlight that Gonzalez and colleagues^25^ previously suggested to adjust calf circumference by subtracting from the measure 3, 7, or 12 cm in case of BMI 25–29 kg/m^2^, 30–39 kg/m^2^, and ≥40 kg/m^2^, respectively. To avoid that the device misclassify or misdiagnose overweight or obese participants, the correction values for such subjects will be clear addressed in the instruction manual. In practice, the assessor can remove the tape from the calf and slide the indicator subtracting the correction centimetres based on BMI. Alternatively, reference charts can be used.

Apart from these limitations, the Lookup 7+ project had offered the unique opportunity to measure calf circumference in a large and relatively unselected population across a wide age spectrum.^25^ The normative curves and centile values generated by our study may greatly facilitate the practical appraisal of muscle mass and allow the timely identification of people with or at risk for malnutrition and sarcopenia.^28^, ^29^, ^30^, ^31^, ^32^ The developed device (calf circumference tape) can represent a useful, easy, quick, inexpensive, and always available tool (to be worn in a lab coat) for a routine assessment of muscle mass in all healthcare settings. In the near future, it will be crucial to compare data from our Italian sample with data from other countries and with data considering other races. Starting from this tool, it will also be possible to ‘modify’ the cut‐offs marking the different risk areas (yellow, orange, and red) and ‘adjust’ these cut‐offs with new and validated values for subjects with BMI above normal.

## Conflict of interest

None declared.

## Funding

The study was funded by the Italian Ministry of Health ‐ Ricerca Corrente 2023 and European Union ‐ Next Generation EU (AGE‐IT). Carni Sostenibili, Danone Italia, Errekappa, Ferrarini, GSK, Italfarmaco, Italia Longeva, IVSI, Laborest, Yakult, Marche Region, Nutricia, PharmaG, ProAction, Professional Dietetics, Serenissima, and Novartis supported the Lookup 7+ project.
